# Veni, vidi, vici? Future spread and ecological impacts of a rapidly expanding invasive predator population

**DOI:** 10.1002/ece3.10728

**Published:** 2023-11-20

**Authors:** David R. Nelsen, Aaron G. Corbit, Angela Chuang, John F. Deitsch, Michael I. Sitvarin, David R. Coyle

**Affiliations:** ^1^ Biology and Allied Health Southern Adventist University Collegedale Tennessee USA; ^2^ Department of Entomology and Nematology University of Florida Lake Alfred Florida USA; ^3^ Ecology and Evolutionary Biology The University of Texas at El Paso El Paso Texas USA; ^4^ Unaffiliated Malta New York USA; ^5^ Forestry and Environmental Conservation Clemson University College of Agriculture Forestry and Life Sciences Clemson South Carolina USA

**Keywords:** Araneae, biodiversity, invasive species, native species, orb weaver, range expansion, species distribution model, *Trichonephila*

## Abstract

Economic and ecological consequences of invasive species make biological invasions an influential driver of global change. Monitoring the spread and impacts of non‐native species is essential, but often difficult, especially during the initial stages of invasion. The Jorō spider, *Trichonephila clavata* (L. Koch, 1878, Araneae: Nephilidae), is a large‐bodied orb weaver native to Asia, likely introduced to northern Georgia, U.S. around 2010. We investigated the nascent invasion of *T. clavata* by constructing species distribution models (SDMs) from crowd‐sourced data to compare the climate *T. clavata* experiences in its native range to its introduced range. We found evidence that the climate of *T. clavata*'s native range differs significantly from its introduced range. Species distribution models trained with observations from its native range predict that the most suitable habitats in North America occur north of its current introduced range. Consistent with SDM predictions, *T. clavata* appears to be spreading faster to the north than to the south. Lastly, we conducted surveys to investigate potential ecological impacts of *T. clavata* on the diversity of native orb weaving spiders. Importantly, *Trichonephila clavata* was the most common and abundant species observed in the survey, and was numerically dominant at half of the sites it was present in. Our models also suggest that there is lower native orb weaver species richness and diversity closer to where *T. clavata* was initially found and where it has been established the longest, though human population density complicates this finding. This early study is the first to forecast how widely this spider may spread in its introduced range and explore its potential ecological impacts. Our results add evidence that *T. clavata* is an invasive species and deserves much more ecological scrutiny.

## Graphical abstract

The Joro spider, *Trichonephila clavata* (L. Koch, 1878, Araneae: Araneidae), is a large‐bodied orb weaver native to Asia, likely introduced to northern Georgia, U.S. around 2010. We found evidence that the climate of *T. clavata*'s native range differs significantly from its introduced range and that the most suitable locations in North America occur to the north of its current introduced range, suggesting its continued ability to spread. Our results also found that *T. clavata* is the most common species encountered, the most numerically abundant species where it is common, and suggest that there is lower native orb weaver species richness and diversity closer to where *T. clavata* was initially found and where it has been established the longest.

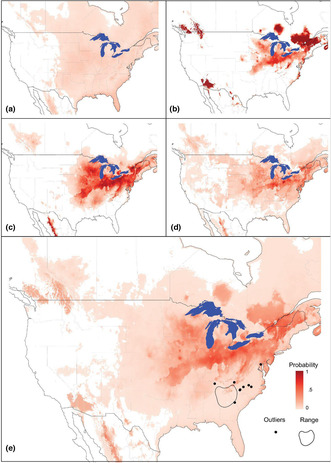

## 1 INTRODUCTION

Biological invasions are one of the most influential drivers of global change, with economic costs rivaling natural disasters (Turbelin et al., 2023). Across all biomes and systems, invasive flora and fauna can directly and indirectly affect native species, change population phenotypic and genotypic diversity, disrupt trophic networks, alter ecosystem function, drive species extinctions, and change the evolutionary trajectory of species (Blackburn et al., 2019; Brooks et al., 2004; Kenis et al., 2009; Ricciardi et al., 2013; Vitousek, 1990). These complex and often significant impacts necessitate efforts toward early detection, evaluation, and if necessary, management of non‐native species. Established monitoring programs exist for some non‐native pest groups (e.g., Rabaglia et al., 2019) but there is no standardized method of population monitoring for most invasive species.

Monitoring the spread and impacts of non‐native species is essential, but often difficult, because their effects on ecosystems may be weak at low initial population densities (Spear et al., 2021). Furthermore, many invasive species may be inconspicuous at low densities, leading to a significant time lag between species establishment and detection. By the time these species are detected, eradication and management options may be highly limited, though this can vary from one ecosystem to the next (Strayer, 2020). When a conspicuous non‐native species is detected early after introduction, it can provide a unique opportunity to evaluate its impacts as it spreads across the landscape.

**FIGURE 1:**
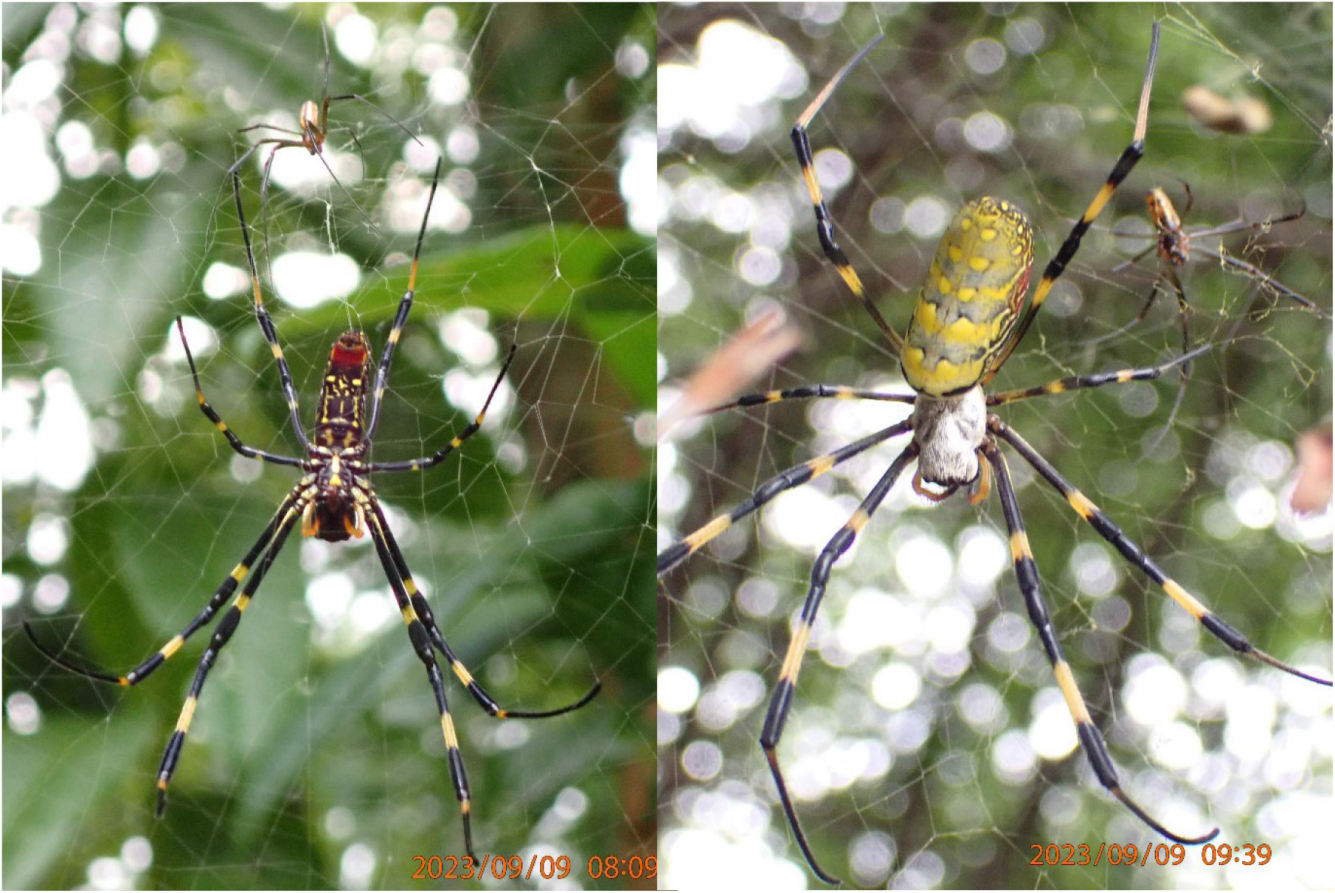
Male and female Jorō spider, *Trichonephila clavata*. On the left is a ventral view of the female while the right shows a dorsal view. Photo credit: David R. Nelsen.

The Jorō spider, *Trichonephila clavata* (L. Koch, 1878, Araneae: Nephilidae; Figure 1), is a large‐bodied orbweaver native to Asia, likely introduced to northern Georgia, United States, around 2010 (Chuang et al., 2023; Hoebeke et al., 2015). The exact method of introduction is unknown but their arrival as stowaway hitchhikers on international shipping cargo is the most likely scenario (Hoebeke et al., 2015). Jorō spiders have now spread into at least four states in the southeastern United States, with breeding populations likely occurring in several additional states (Chuang et al., 2023, www.inaturalist.org). The rate at which these non‐native arthropods are spreading appears to be increasing exponentially (Chuang et al., 2023), following a lag time typical of invasive species (Sakai et al., 2001), with their presence in at least ~120,000 km^2^ as of summer 2022 (Chuang et al., 2023).

There is widespread interest and speculation on how far *T. clavata* may spread across North America. Given how recently this species was introduced to the continent, its native range may provide important clues to its future potential geographic distribution. *Trichonephila clavata* inhabits more northern latitudes in its native range than in its introduced range, which supports the potential for further northward spread. Despite the potential limitation of transferability between a species native and introduced ranges (Liu et al., 2020), species distribution models (SDMs) can be a useful tool for providing future range predictions for invasive species and optimizing control and management strategies (Giljohann et al., 2011; Jiménez‐Valverde et al., 2011; Kramer et al., 2017; Tingley et al., 2018; Tulloch et al., 2015). These models can utilize opportunistic occurrence data, which can be derived from public monitoring efforts.

The sudden ubiquity of *T. clavata* in the southeastern United States coupled with their large size, bright coloration, and propensity to dwell around homes and other structures has brought these non‐native spiders to the forefront of public awareness. In particular, the crowdsourced biodiversity observation site, iNaturalist (www.inaturalist.org), has been used as a platform for community and professional scientists, naturalists, and laypeople to upload photographs, verify identifications, and map distributions of several non‐native pests worldwide (e.g., Della Rocca & Milanesi, 2022; Martel et al., 2022; Werenkraut et al., 2020). There are over 6700 “research grade” observations of *T. clavata* globally as of May 2023 (i.e., including a photograph and multiple user verifications of species identity), nearly half of which are from the non‐native range. These observations first allowed us to make preliminary estimates of its range boundaries (Chuang et al., 2023), and here we further use them to construct a SDM to determine future range predictions and assess how it compares to *T. clavata*'s current range expansion rate.

*Trichonephila clavata*'s large size may facilitate detection, which can aid in monitoring its ecological impacts as it spreads, despite the relative recency of invasion. Although invasive spiders have historically received little attention, case studies have demonstrated the ability of certain invasive spider species to compete with, displace, and prey on native spiders (Bednarski et al., 2010; Coticchio et al., 2023; Houser et al., 2014; Nyffeler et al., 1986), which can shift spider community composition (Jakob et al., 2011). *Trichonephila clavata* has been observed in locally high abundances, which may limit available web spaces for native orb weavers. At longer‐established sites in Georgia, *T. clavata* has seemingly become the most common web‐building spider (Davis & Frick, 2022; David R. Coyle, David R. Nelsen, John F. Deitsch, Michael I. Sitvarin, personal observations). This raises questions of whether these densities come at the cost of native orb weaver abundance and diversity, especially with the native congener *Trichonephila clavipes* (Linnaeus, 1767).

We address standing questions on the nascent invasion of *T. clavata* in this study by (1) building SDMs based on environmental features of their native range to predict the potential future spread of *T. clavata* in North America; (2) using SDM predictions to inform current range expansion dynamics; (3) using transect survey data to determine the effects of *T. clavata* presence on the abundance and diversity of orb weaving spiders in a portion of the southeastern United States.

## 2 METHODS

### 2.1 Objective 1

Building SDMs based on environmental features of *T. clavata*'s native range to predict the potential future spread in North America.

#### 2.1.1 Data and data cleaning

We downloaded all available research‐grade records of *T. clavata* observations from both iNaturalist and GBIF as of November 10, 2022. While many of the research‐grade observations on iNaturalist from North America have been verified by one or more authors, we did not do so for Asian records. However, recent research has suggested that research‐grade observations are robust to scrutiny, especially for large, colorful, and conspicuous species like *T. clavata* (Campbell et al., 2023). We split these data into two regions: Asia and North America. We also downloaded the bioclimate dataset (19 variables) and wind speed from the WorldClim database (version 2.1) at a resolution of 2.5 arc‐minutes (~4 km) (Fick & Hijmans, 2017) and refer to these as bioclimate predictors hereafter. We included wind speed as a bioclimate predictor for a couple of reasons. First, previous research on another air‐dispersing spider species used windspeed as a predictor for their SDMs (Segura‐Hernández et al., 2023). Second, we felt that while wind direction may be more relevant to understanding the patterns of range expansion we currently observe with *T. clavata* (research objective 2), wind speed is a more relevant indicator of habitat suitability, given its effect on web architecture (see discussion in Tew & Hesselberg, 2017). We calculated the maximum wind speed variable based on the wind speed variable provided by WorldClim, following Segura‐Hernández et al. (2023). We used QGIS to trim the original bioclimate predictor files to an Asian (52.083, −7.042: 146.833, 52.375) and North American (−125.042, 23.833: −64.417, 61.792) extent, respectively. We filtered Asian *T. clavata* observations to only allow one observation per 4 km^2^ grid (spatial thinning) to control for biased sampling (Kiedrzyński et al., 2017; Steen et al., 2021) inherent in community‐science data. We also randomly selected 50,000 background points (Valavi et al., 2022) for both Asia and North America and, along with our reduced observations, created two datasets that included the bioclimate predictors at each location.

#### 2.1.2 Comparison of Asian and North American bioclimate predictors

We compared the climate (20 bioclimate predictors) of *T. clavata*'s native range to its introduced range using box plots, Mann–Whitney *U* tests, and the associated rank biserial correlation measure of effect size.

#### 2.1.3 Bioclimate predictor reduction

We first tested the predictive value of each bioclimate predictor using bivariate logistic regression models. We then ranked each predictor based on the odds ratios derived from the models. Correlation analysis was then used to determine the degree to which each bioclimatic predictor was correlated with all other predictors. Highly correlated pairs of predictors (Pearson's *r* > .7) were then identified. An iterative process was then used to sequentially remove the lowest performing predictors involved in any highly correlated pair until none were left. This reduced our bioclimate predictor dataset from 20 (19 bioclimate variables and wind speed) to five: minimum temperature of the coldest month, mean temperature of the wettest quarter, precipitation seasonality (coefficient of variation), precipitation of warmest quarter, and wind speed.

#### 2.1.4 Creation of species distribution models

After centering and scaling each predictor, we utilized four methods to create species distribution models trained on the Asian data. We used two high‐performing machine learning approaches (a down‐sampled random forest and Maxent; Liaw & Wiener, 2002, Hijmans et al., 2011), and two statistical approaches (GLM and GAM; Wood, 2017). Because each SDM approach has its strengths and limitations, we followed the suggestions of Zhu et al. (2020) and Valavi et al. (2022). We selected well‐established methods that performed well and that, when combined (i.e., averaged), may help account for each model's biases. Valavi et al. (2022) provide a comprehensive evaluation of many SDM methods, and we have followed their suggestions closely. We used the default settings for Maxent, as they have been shown to perform well compared to models with tuned complexity for a recent study on a large dataset (Valavi et al., 2022). Concerning our random forest model, we translated the hyperparameter settings described by Valavi et al. (2022) for their best‐performing random forest: in our case, ntrees = 1000, mytr = sqrt(5), sampsize = 51,269. Lastly, we selected 50,000 background points, again based on Valavi et al. (2022; see their discussion on selecting background points), and we selected these points from the same geographic extent described for our Asian bioclimate predictor. Model creation was done using the *mgcv* (version 1.8‐42, GAM and GLM), *dismo* (version 1.3‐14, MaxEnt), and *randomForest* (version 4.7‐1.1) packages in R. Data, R scripts, and other associated files can be found at 10.5281/zenodo.8091991. To assess model overfitting, we performed 10‐fold cross‐validation on each of the four model types, reporting area under the curve (AUC) and mean absolute error (MAE) following the suggestion of Konowalik and Nosol (2021). Because conventional random cross‐validation methods can underestimate prediction error for spatial data, we used the *blockCV* package (version 3.1‐3) to assign folds using the default random spatial blocking technique and a block size of 860 km (Valavi et al., 2019). We then used all available data to construct the final models. We used the predictions from these four methods and created an averaged model using QGIS (version 3.26.3). We report all four SDMs and the averaged model in the results to show their variation.

In addition to the models trained on the Asian observations, we also trained a Maxent model using observations from the introduced range; however, due to the highly clustered nature of these observations around Atlanta, GA, and because *T. clavata* has continued to expand its range each year, we felt that this model did not generalize well or add significant new insights. Thus, we do not report these results here.

### 2.2 Objective 2

Evaluation of current range expansion dynamics.

#### 2.2.1 Distance (Method 1)

We calculated the distance of every North American *T. clavata* record from the centroid of invasion. We calculated the invasion centroid by averaging the nine longitude and latitude coordinates from Hoebeke et al. (2015), which provides the first documented observations of *T. clavata* within the United States. We classified every observation as being NE, SE, NW, or SW from the centroid of invasion. To approximate the leading edge of *T. clavata* range expansion, we selected the three farthest observations in each direction from the centroid each year from 2018 to 2022. We calculated the mean value of the three selected observations and used this as a measure of the distance of range expansion (km) in each direction. [There was only one observation in 2018 in the SE direction.]

#### 2.2.2 Area (Method 2)

We calculated the area (km^2^) in North America occupied by *T. clavata* in each year from 2018 to 2022 using kernel density estimation (KDE) via the *amt* R package (version 0.2.1.0, Signer et al., 2019). To control for spatial bias in iNaturalist observations from North America (i.e., observations are more heavily focused near large cities), we spatially thinned *T. clavata* observations within each year using the same procedure described above. We performed KDE at 0.99 isopleth level at the reference bandwidth (*h* = (0.5*(sd long + sd lat)) * *n* ^‐⅙^). We then overlaid each year's range on to the four quadrants (NE of centroid, SE of centroid, NW of centroid, and SW of centroid) and calculated the area (km^2^) of *T. clavata*'s range in each of these four directions from 2018 to 2022.

### 2.3 Objective 3

Determining the effects of *T. clavata*'s presence on the abundance and diversity of orb weaving spiders with transect surveys.

#### 2.3.1 Transect data collection

**FIGURE 2:**
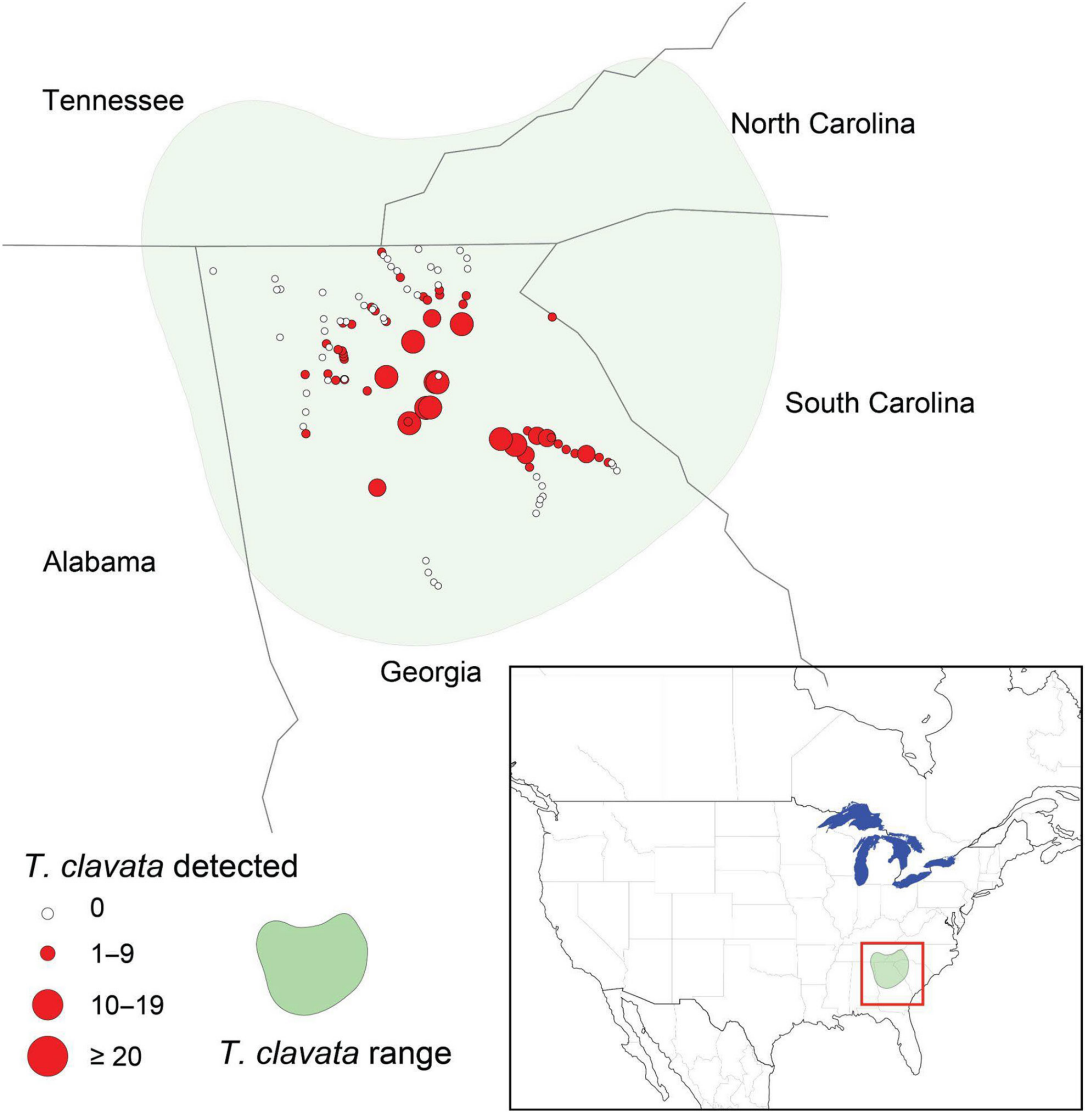
Map of survey transect locations where *T. clavata* and native orb weaver abundance was recorded. Abundance of *T. clavata* is indicated by the size and color of the circle. Range of *T. clavata* (using iNaturalist data through December 2022) is indicated by the shaded region. Inset map shows the location of *T. clavata* range and survey transects in relation to North America.

Between August 29 and November 8, 2022, we surveyed 103 locations across northern Georgia, United States (Figure 2). Peak observations of *T. clavata* reported to iNaturalist occur during this period as do many of the species that we encountered during the survey. We started a transect either at a location where *T. clavata* had never been observed previously or an area where *T. clavata* had been observed for at least three consecutive years. We surveyed in publicly accessible locations such as parks, trailheads, and forest edges by the sides of roads. All researchers followed the same survey protocol. We used a method similar to a Pollard walk, as visual census techniques have been shown to be effective for counting spiders with conspicuous webs (Lubin, 1978). We included all spider species that make orb webs in our survey, including those in the families Araneidae, Tetragnathidae, Uloboridae, and the disputed family Nephilidae (Kulkarni et al., 2023; Kuntner et al., 2023), which includes *T. clavata* and its native congener, *T. clavipes*. We first located and photographed an orb weaving spider. We then started a 10‐min timer, during which we moved continuously at a slow pace, recording the presence and abundance of all orb weaving spider species, pausing to photograph each individual to confirm their initial identification. We recorded the date, time, temperature, windspeed, and amount of precipitation within the last 24 h at each survey location.

#### 2.3.2 Variable calculation

Following the recommendations of Mouillot and Lepretre (1999) and Morris et al. (2014) we used several measures of species diversity, including species richness, Shannon–Weaver's index, and Simpson's diversity index as our response (dependent) variables. We used the vegan package (version 2.6‐4) in R (Oksanen et al., 2022) to calculate Shannon's and Simpson's diversity indexes. *Trichonephila clavata* was much more abundant than native orb weaving species across many locations, especially those closest to the invasion centroid. This, coupled with the fact that Shannon's and Simpson's indexes consider both the number of species observed and their evenness, we calculated Shannon's and Simpson's indexes independent of *T. clavata*. We did this to ensure that comparisons across our transects were not affected by the high density of *T. clavata* in some locations, allowing us to see if changes in diversity were not just a result of changes in evenness.

For our predictor (independent) variable, we estimated two measures of the historic presence of *T. clavata* across our survey area: (1) distance from the invasion centroid to each surveyed location (described above) and (2) an estimate of how long *T. clavata* has been present at a surveyed location. We calculated the number of years *T. clavata* had been in an area by creating a 10 km by 10 km grid, noting the date of the first observation within each cell, and then calculating the number of years from that time until November 2022. We assigned a value of 1 year to cells with a first observation in 2022, to account for the presence of *T. clavata* during the summer of 2022 prior to field surveys. We assigned values to survey locations according to the grid cell within which they are located. Lastly, because the introduction of *T. clavata* occurred around the greater Atlanta metropolitan area, we controlled for urbanization by obtaining the human population density of each surveyed location from the “Gridded Population of the World (GPW), v4” dataset (CIESIN, 2018) at a resolution of 30 arc‐seconds (1 km).

#### 2.3.3 Data analysis

We tested for spatial autocorrelation between survey locations using the *spdep* package (version 1.2‐8) in R (Bivand, 2022; Bivand et al., 2013). We constructed a spatial correlogram to visualize and explore our data. We also calculated Moran's I on model residuals for our three diversity indexes using two approaches. First, we ensured that all locations had at least one neighbor. Second, we applied a 10 km radius around each location; all locations within that 10 km radius were considered neighbors. The spatial correlogram showed no clear spatial pattern and none of the Moran's I results were significant. Following these results, we used both linear and generalized linear models to statistically test for the impact of *T. clavata* presence on the species richness and diversity of native orb weaving spiders.

To aid in the construction of biologically driven statistical models, we a priori constructed a directed acyclic graph (DAG) to represent our proposed causal relationship between the predictor and response variables, illustrate potential confounders and selection bias, and identify strategies to deal with them (Greenland et al., 1999; Greenland & Pearl, 2014; Figure S1 in the Appendix S1). Directed acyclic graphs do this by graphically depicting the hypothesized connections between variables (nodes) via arrows (edges). Concerning the identification of confounding, any variable that points to both the predictor and response variables is considered a confounder and should be adjusted for either experimentally or as part of the analysis. Although DAGs are very useful in model creation, they are of limited use for determining which interactions to include in the final models (Attia et al., 2022). Therefore, for each response variable we created several models, some with only main effects and some including interactions. We evaluated model performance using ΔAIC_c_ values calculated using the AICcmodavg package (version 2.3‐2) in R (Mazerolle, 2023). We limit our results and discussion to the best‐performing models with species richness and Shannon's index as the response variables. We used multivariate linear regression for Shannon's index, Simpson's index, and multivariate Poisson regression when species richness was the response variable. Overall, the best‐performing model equations followed the format:

$$\begin{array}{c} \text{Response variable}\sim \text{Temp}\;\left(C{}^{\circ}\right)\\ +\text{Windspeed}\;\left(\mathrm{kph}\right)+\text{Rain in the last}\;24\;h\;\left(\mathrm{cm}\right)\\ +\text{Julian days}\;\left(\text{out of}\;365\right)\\ +\text{Human population}\;{\text{density}}^{*}\text{Predictor variable of interest}, \end{array}$$

where the response variable was either Shannon's index, Simpson's index, or species richness, and the predictor variable of interest was the distance from the invasion centroid or years *T. clavata* had been observed in that area. Table S1 in the Appendix S1 contains the results for all models and their respective ΔAIC_c_ scores. We did not include time of day in the model as we routinely found webs or web‐remnants and were able to locate the occupant even if it was not visible within its web.

To avoid the “table 2” fallacy, that is, reporting effect estimates for all variables included in the model and potentially contributing to their misinterpretation, we only report the model coefficients for our interaction term and not for any of the other variables included to adjust for potential confounding (Westreich & Greenland, 2013).

Lastly, we only present the graphical results for species richness and Shannon's index in the main manuscript. We did this because Shannon's index and Simpson's index measure similar aspects of diversity, our analyses found that the direction of changes in these indexes were the same (see Figure S2 for Simpson's index in the Appendix S1), and a four‐paneled figure was easier to read.

## 3 RESULTS

### 3.1 Comparison of Asian and North American bioclimate predictors

**FIGURE 3:**
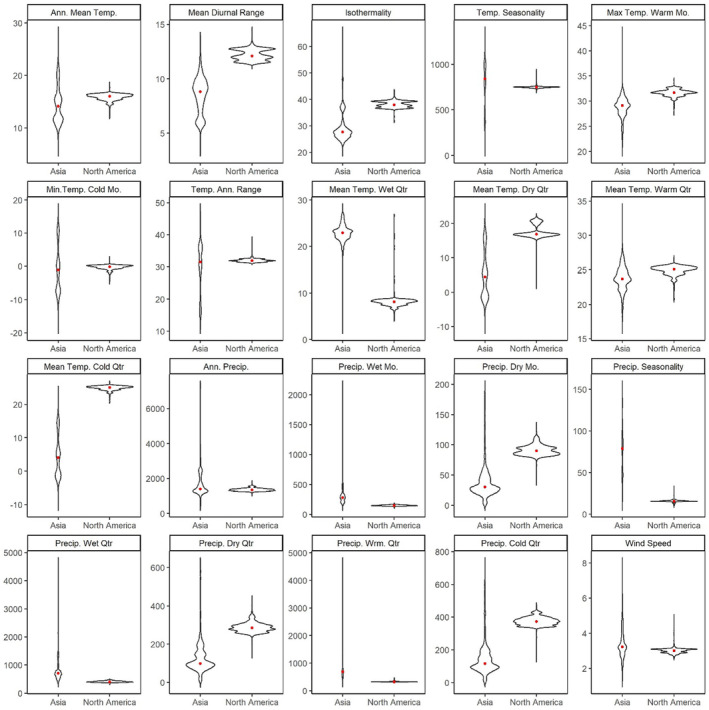
A comparison of the values of the 19 standard bioclimatic variables and wind speed for each of the known ranges of *Trichonephila clavata* in both Asia and North America. Red dot = median. Units for temperature‐related variables are in °C except for *Temp. Seasonality* which is in °C·100. The unit for precipitation variables is kg m^−2^. Wind speed is in m s^−1^. Spider observations from iNaturalist (https://www.inaturalist.org/) and bioclimatic data from WorldClim (https://worldclim.org/).

All 20 predictor variables differed significantly between Asian and North American windows (Figure 3, Table S2 in the Appendix S1), as most climate predictors showed a large effect (*r*_rb_ > .5) for differences by region. However, the mean temperature of the warmest quarter, temperature seasonality, annual mean temperature, and wind speed showed only moderate effects (*r*_rb_ > .3), and minimum temperature of coldest month, annual precipitation, and annual temperature range showed small effects (Figure 2, Table S2 in the Appendix S1).

These results show that for climate variables like those related to precipitation, total annual precipitation between known *T. clavata* locations in Asia and North America differs substantially less than the pattern of when the precipitation falls. Asian *T. clavata* experiences a greater seasonal fluctuation in rainfall than those in North America (i.e., Asian spiders experience more distinct rainy and dry seasons). Likewise, temperature seasonality and the annual temperature range show less of a difference than how the temperature fluctuates throughout the year. North American spiders experience greater daily fluctuations in temperature (max temperature in the warmest month and mean diurnal range) and are warmer in the coldest quarter and driest quarters, while Asian spiders experience warmer conditions in the wettest quarter.

### 3.2 Species distribution models

**FIGURE 4:**
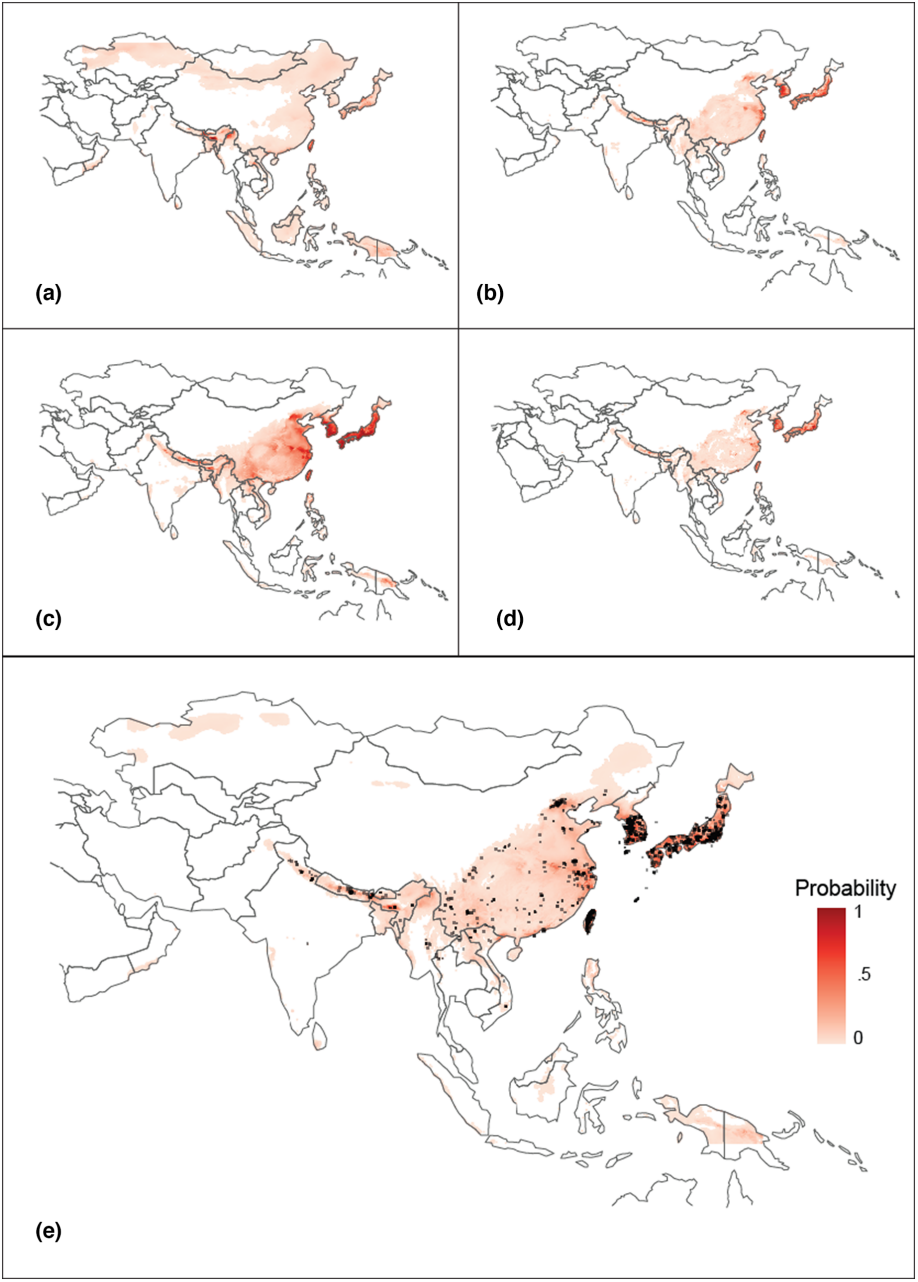
Species distribution model (SDM) of *Trichonephila clavata*'s native range trained on Asian observations as of December 2022: (a) General Linear Model: cross‐validation Mean AUC_ROC_ = 0.77 and MAE = 0.049, (b) General Additive Model: cross‐validation Mean AUC_ROC_ = 0.92 and MAE = 0.038, (c) Maxent: Mean AUC_ROC_ = 0.92 and MAE = 0.062, (d) Random Forest: Mean AUC_ROC_ = 0.92 and MAE = 0.039, and (e) Averaged prediction of all four models, calculated as an equally weighted average across all four primary models.

Our species distribution models trained on Asian occurrences are shown in Figure 4, with their respective performances (AUC and MAE). Ten‐fold cross‐validation results ranged from Mean AUC_ROC_ = 0.77–0.92 and Mean MAE = 0.038–0.062, with the GLM having the lowest AUC score.

**FIGURE 5:**
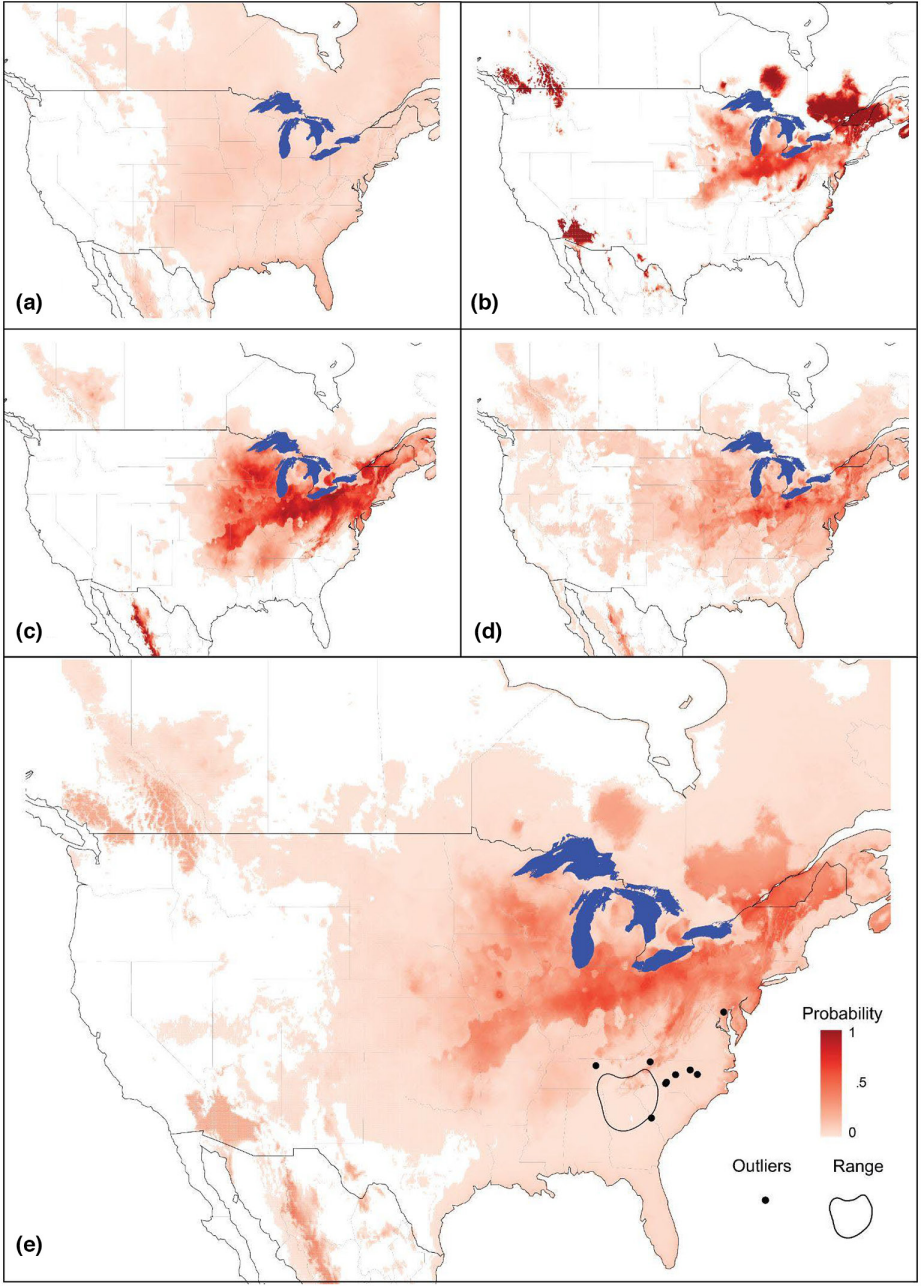
Species distribution model (SDM) predictions of *Trichonephila clavata*'s suitable range in North America based on its native Asian distribution: (a) General Linear Model, (b) General Additive Model, (c) Maxent, (d) Random Forest, and (e) Averaged prediction of all four models, calculated as an equally weighted average across all four primary models, including *T. clavata's* introduced range as of December 2022.

Overall, *Trichonephila clavata* has become established in the southeastern United States, and our SDMs do not predict that the habitat in this region as highly suitable for *T. clavata*. However, our SDMs suggest that areas further north have greater habitat suitability for *T. clavata*. Our application of these models onto North America suggests that the Great Lakes region of the United States and Canada extending throughout the midwestern and northeastern United States, and into eastern Canada, are potentially suitable for future range expansion (Figure 5). Our models also show that some areas of the American northwest (both the United States and Canada, all four models) and the mountainous region of northwestern Mexico (GLM, MaxEnt, and RandomForest, but not the GAM) may also be favorable for *T. clavata* Taken together, our ensemble (averaged) model suggests that *T. clavata* may be capable of surviving in a substantial portion of North America, from its current distribution west to the Great Plains and north into Canada, and in areas of the Pacific Northwest in the United States and Canada.

### 3.3 Range expansion dynamics

**FIGURE 6:**
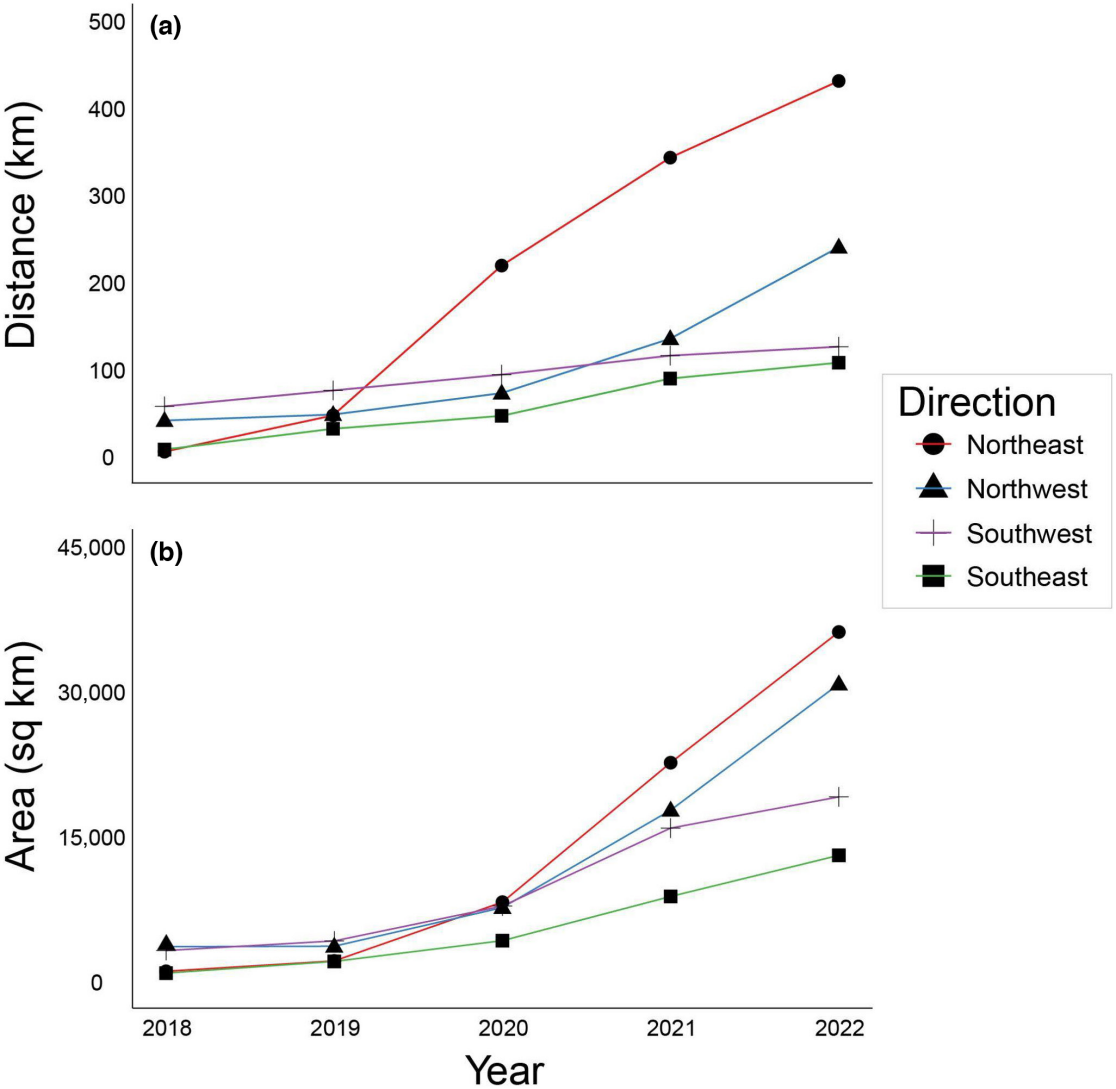
Rate of range expansion by *Trichonephila clavata* from 2018 through 2022, measured by (a) distance (km) of range edge from point of introduction and (b) area (km^2^).

We evaluated the current range expansion dynamics of *T. clavata* as captured by iNaturalist data. Whether quantified by area or distance, the data indicate that *T. clavata* is spreading faster to the north than to the south (Figure 6). *Trichonephila clavata* has spread 436 and 245 km to the NE and NW, respectively, compared to 113 and 131 km to the SE and SW.

### 3.4 Descriptive results of the survey

**FIGURE 7:**
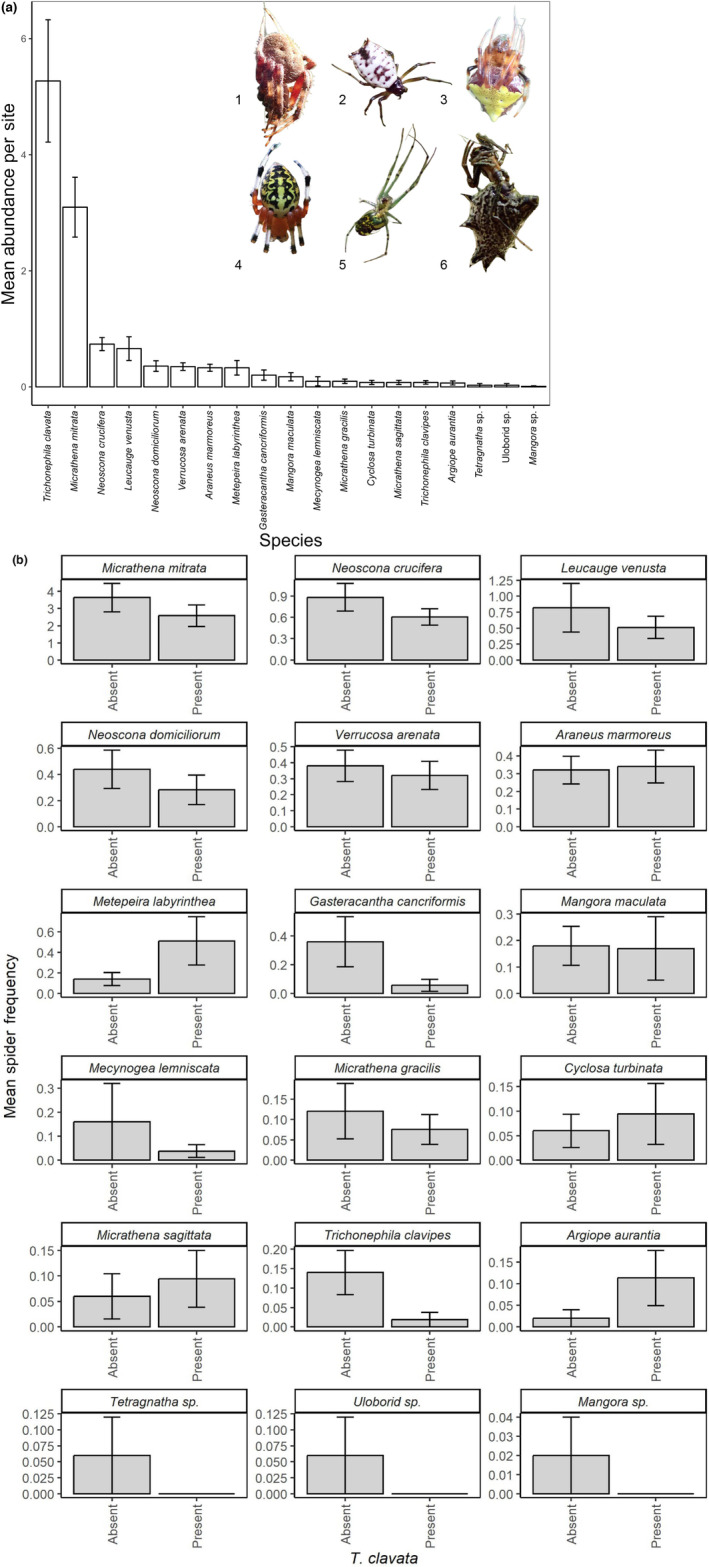
Species observed (*N* = 19) during survey of 103 locations across an area of 32,600.18 km^2^. (a) Mean abundance (±SE) of all species. (b) Mean abundance (with 95% CI) grouped by the presence/absence of *Trichonephila clavata* at the survey location. Species images: (1) *Neoscona crucifera*, (2) *Micrathena mitrata*, (3) *Verrucosa arenata*, (4) *Araneus marmoreus*, (5) *Leucauge venusta, and* (6) *Micrathena gracilis*. Photo credit: David R. Nelsen (images 1, 2, 3, 5, and 6) and John F. Deitsch (image 4).

We found 19 different orb weaving spider species on our transect surveys, though spider abundance was numerically dominated by *T. clavata* and the native *Micrathena mitrata* (Figure 7; Table S3 in the Appendix S1). *Trichonephila clavata* (n = 543) was observed at the most locations (53 of 103, or 51.5%), was the most abundant spider observed on average (>5 individuals per location on average) and was the species with the most individuals found in total across all surveyed locations. At half of the sites where it was present, *T. clavata* was the most numerically dominant species. *Micrathena mitrata* (*n* = 319) was the next most abundant, found in 49 of 103 locations (47.6%) with an average of >3 individuals per site. Only one other species (*Neoscona crucifera*) was observed at >40% of surveyed locations (44 of 103 or 42.7%), and it, along with all other species, was less abundant (<2 individuals on average per site) overall. The total number of individuals observed for all species other than *T. clavata* and *M. mitrata* (*n* = 386) was less than *T. clavata* alone and only slightly larger than *M. mitrata*.

### 3.5 Impact of *T. clavata* on native orb weaving spider species

We found evidence that areas where *T. clavata* has been present longer, that is, observed for more years and closer to the invasion centroid, had significantly lower diversity of orb weaving spiders (Table 1).

**TABLE 1:** Results of linear and generalized linear models testing if the historic presence of *Trichonephila clavata* affects the abundance and diversity of native orb weaving species.

Response	Historic predictor	Coefficient ± SE	*p*
Species Richness	Distance from Centroid	2.9E‐8 ± 1.3E‐8	.032
	Years at Location	−6.6E‐4 ± 2.8E‐4	.016
Shannon's	Distance from Centroid	2.1E‐8 ± 1.0E‐8	.039
	Years at Location	−4.0E‐4 ± 1.8E‐4	.033
Simpson's	Distance from Centroid	9.2E‐9 ± 4.5E‐9	.043
	Years at Location	−1.7E‐4 ± 8.0E‐5	.034

*Note*: Model equation: Diversity variable ~ Temp (C°) + Windspeed (kph) + Rainfall in the last 24 h (cm) + Julian days (out of 365) + Human population density*Predictor variable. Distance coefficients are in meters.

**FIGURE 8:**
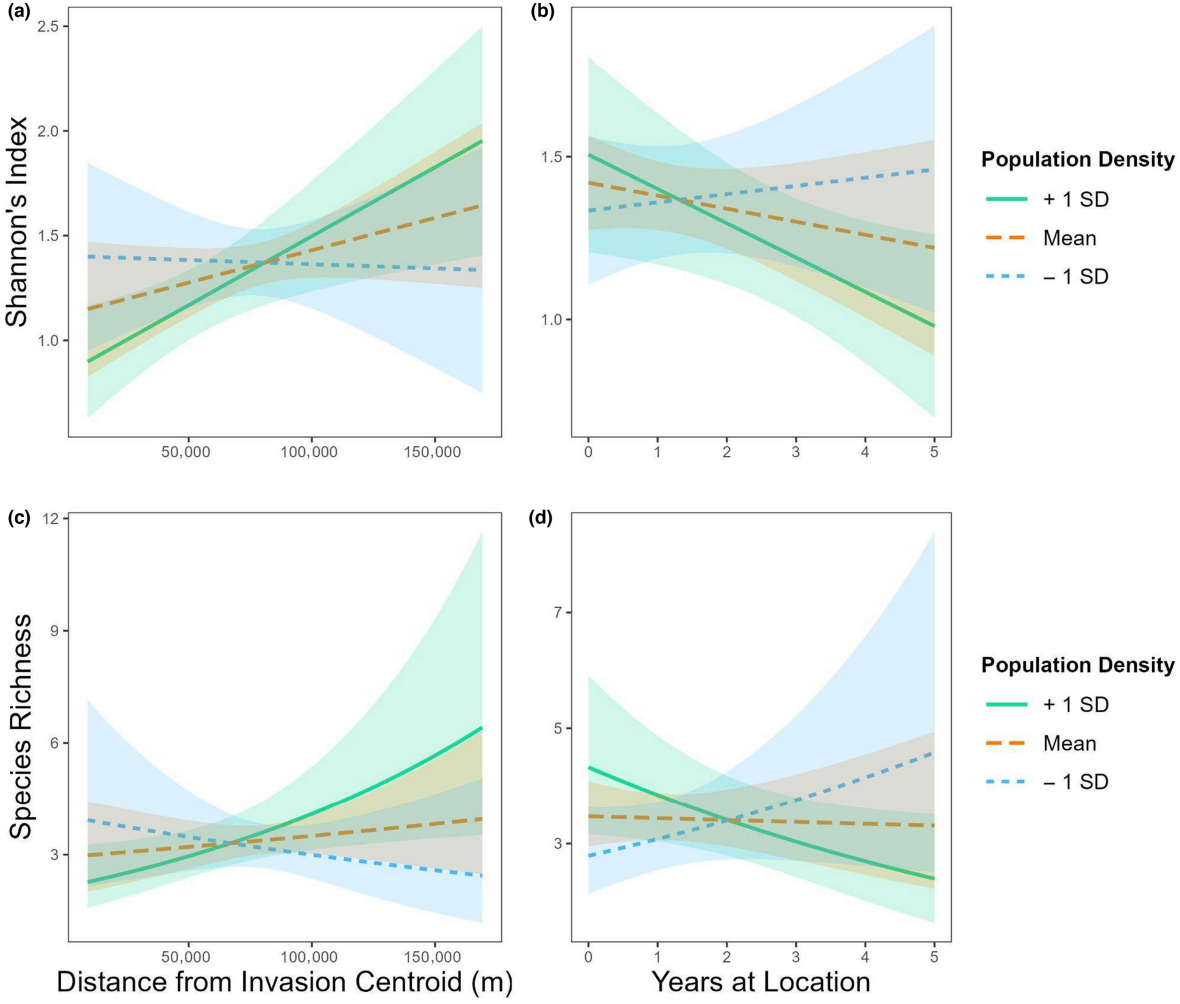
Interaction plots based on linear and generalized linear regression models of a location's human population density and a measure of *Trichonephila clavata'*s presence (in space or time) on orb weaving spider diversity: (a) response variable = Shannon–Weaver's index and predictor variable = Distance from invasion centroid, (b) response variable = Shannon–Weaver's index and predictor variable = Years *T. clavata* have been observed in that area, (c) response variable = Species richness and predictor variable = Distance from invasion centroid, and (d) response variable = Species richness and predictor variable = Years *T. clavata* have been observed in that area. Shading around trend lines represents 95% CI.

Model interactions suggest that the effect of *T. clavata* may be stronger in areas with higher human population densities, see Figure 8. Our models predict that a site located at the invasion centroid with a high human population density (mean + 1 SD) will have an average of 2.18 species and a Shannon's score of 0.83. In contrast, a site located 100 km away but with all other variables being equal will have a higher species richness of 4.22 and a Shannon's diversity score of 1.50. This results in a difference of 2.04 species and 0.67 difference in Shannon's diversity index. However, the model predictions made for similar sites except for an average human population density (mean) would result in a species richness of 3.00 and a Shannon's diversity score of 1.12 for a site at the invasion centroid and a species richness of 3.60 and a Shannon's index of 1.43 for a site 100 km away. This results in smaller differences in species richness and Shannon's diversity indices, 0.60 and 0.31, respectively.

Models that include the number of years *T. clavata* has been present at a site give similar results. Our models indicate that sites with a high human population density where *T. clavata* has never been present will have an average of 4.46 species and a Shannon's score of 1.51, while a site that had *T. clavata* for 5 years, with all other variables being equal, will have a lower species richness of 2.43 and a Shannon's diversity score of 0.98 which results in a species richness difference of 2.03 and a Shannon's diversity index difference of 0.53. Comparing this to similar sites but with an average human population density, the model predicts a species richness of 3.58 and a Shannon's diversity score of 1.43 for a site where *T. clavata* has never been present and a species richness of 3.38 and a Shannon's index of 1.22 for a site where *T. clavata* has been present for 5 years. Again, this results in a smaller change in species richness and Shannon's diversity indices, 0.20 and 0.21, respectively.

## 4 DISCUSSION

Our SDMs suggest that much of eastern North America, along with pockets of the West, are climatically suitable for *T. clavata*. This species has expanded its range rapidly since its arrival, with calculations suggesting the fastest spread in the northeast direction. Preliminary surveys of orb weavers both in and beyond *T. clavata*'s current range show a few striking patterns—that *T. clavata* is already both the most common and often the most abundant species at sites, and lower native orb weaver diversity where *T. clavata* has occurred the longest.

### 4.1 Species distribution models

Overall, our results suggest that *T. clavata* may be capable of significant range expansion within North America. Our species distribution models, basing *T. clavata*'s optimal range on its native Asian distribution, found that *T. clavata* may be capable of expanding its range into regions south of the Great Lakes and into all states in the northeastern United States. All four models also predicted portions of Canada, including provinces north of the Great Lakes, such as Quebec and Ontario, as well as more fragmented portions of Alberta and British Columbia as potential habitat. Lastly, the models suggested fragmented sections of Mexico, near Chihuahua, may also be suitable for *T. clavata* continued expansion. These areas are all currently outside of *T. clavata*'s introduced range, though ongoing spread may eventually result in their presence in these distant regions.

While there are differences in the performance and predictions from each SDM, we retained each model, including the more diffuse predictions of the GLM. Because some methods may overpredict while others underpredict (Zhu et al., 2020), we felt the most prudent approach was to combine all models as the “truth” may lie somewhere in between. Concerning the GLM, its inclusion is important for two reasons. First, many areas it identified as suitable correspond well with the random forest predictions. Second, its predicted probabilities were smaller than the other models. Thus, its inclusion tempers the predictions of the other models, and the average model does not retain most of the areas found only in the GLM. Overall, we encourage caution when interpreting predicted regions unique to any specific model. Our GAM and MaxEnt models predict that areas where *T. clavata* is apparently thriving in the southeastern United States are not suitable habitats, while our RandomForest and GLM models predicted these areas as having only low suitability. This may be due in part to the relative differences between the bioclimate predictors for Asia and North America. Not only did many of our predictor variables have a large difference (effect size) between regions, but three (mean temperature of wettest quarter, precipitation seasonality, and precipitation of warmest quarter) of the five variables used in our SDMs had large differences, while the remaining two variables (maximum wind speed and precipitation of warmest quarter) had moderate and small differences, respectively. In their North American range, *T. clavata* experiences lower mean temperatures during the wettest quarter, lower precipitation seasonality, and lower precipitation during the warmest quarter.

All of this suggests that models trained on data from Asia may have limited application when used to predict what habitats in North America could potentially support *T. clavata*'s continued range expansion. However, despite this limitation, these models still offer insights into the possible range expansion of *T. clavata* throughout North America. First, similar to Luo et al. (2022), and following the recommendation of Hui (2023), we limit our interpretation of our model results to predicting the potential distribution of *T. clavata* in a novel range (North America) based on niche space estimate from its native range in Asia. Our results suggest that *T. clavata* may have experienced a shift in its realized niche, similar to what Luo et al. (2022) found with *Latrodectus hasselti* (which is native to Australia) in its introduced range of Japan, New Zealand, and Southeast Asia. Second, although all climatic variables differed significantly between Asia and North America, the effect size differences between minimum temperature, temperature range, and annual precipitation were small or very small (see Table S2 in the Appendix S1). This suggests that areas of North America identified as most suitable for *T. clavata* based on its native Asian range have temperatures and total precipitation that this species is already capable of surviving.

Biotic interactions may explain why *T. clavata*'s latitudinal distribution in Asia differs so much from its current introduced range. Native North American antagonists (e.g., birds, predatory wasps, and parasitoids) may not be regulating *T. clavata* populations as they do in Asia (i.e., the enemy release hypothesis; Roy et al., 2011, but see Powell & Taylor, 2017). Competition should also be considered, but it remains unknown whether smaller orb weavers are significant competitors, as *T. clavata* may capture larger prey that is unavailable to smaller spiders.

**FIGURE 9:**
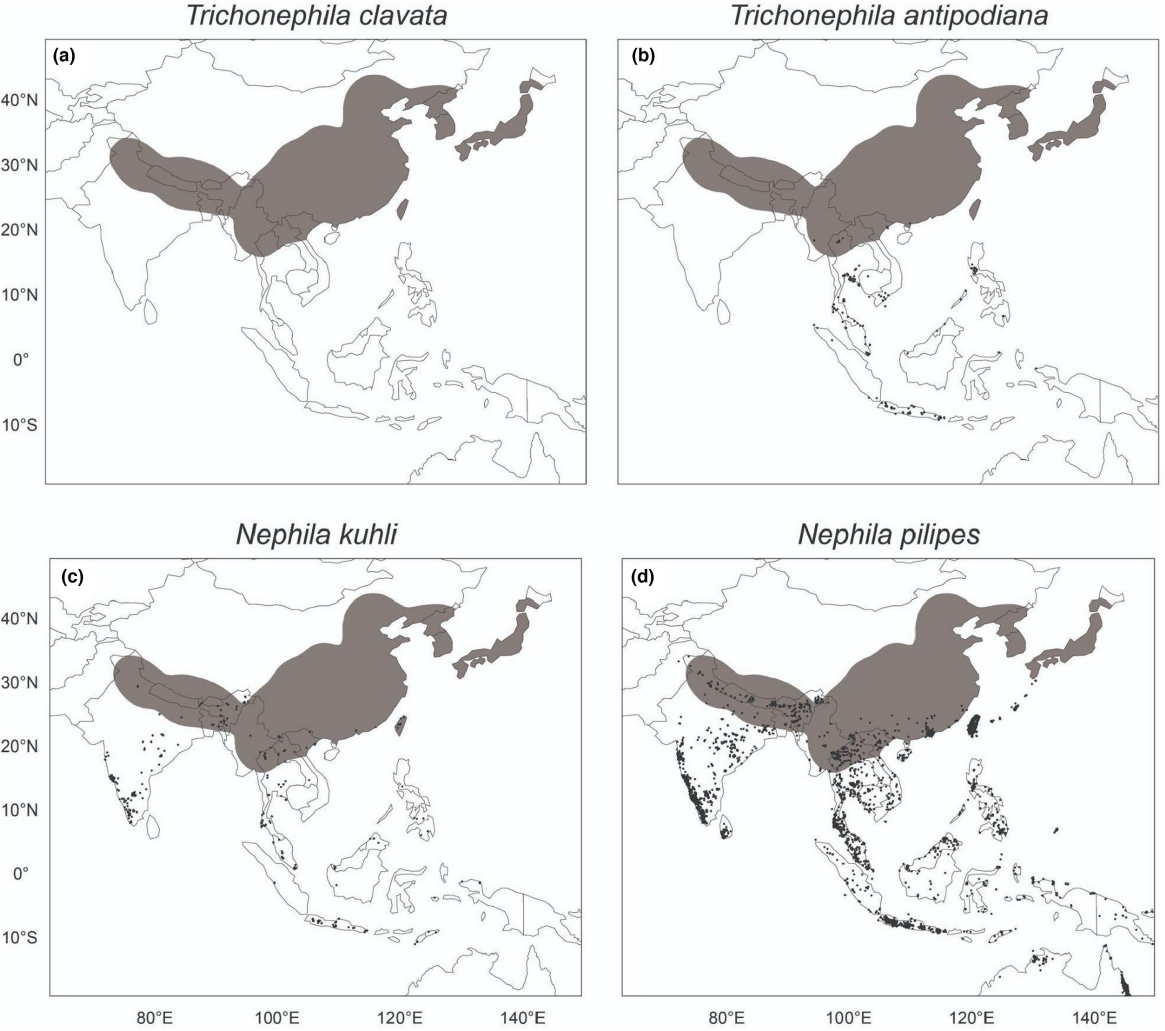
Distribution of (a) *Trichonephila clavata* in its native range in comparison with occurrences of three related species, (b) *Trichonephila antipodiana*, (c) *Nephila kuhli*, and (d) *Nephila pilipes* based on iNaturalist and GBIF records (www.inaturalist.org; www.gbif.org) as of May 2023. Gray shading represents *T. clavata*'s range in all panels. Darker points represent individual reports of other species.

However, competition with other large‐bodied orb weavers with correspondingly large webs (e.g., *Argiope* spp.) is likely, especially between related species in the family Nephilidae that may share ecologically similar niches. *Trichonephila clavata*'s Asian range comprises Japan, the Korean peninsula, China, Taiwan, Myanmar, as well as the Himalayan regions extending to India (Figure 9). While *T. clavata*'s range overlaps with the more geographically common *Nephila pilipes* (Fabricius, 1793), *T. clavata* rarely occurs with *Trichonephila antipodiana* (Walkenaer, 1841) and *Nephila kuhli* (Doleschall, 1859). In fact, *T. antipodiana* and *N. kuhli* are only present where *T. clavata* is absent or rarely found in southern Myanmar, Thailand, Laos, and Cambodia (Figure 8); this pattern is reflected in iNaturalist occurrence data and on GBIF records (www.gbif.org).

This striking lack of overlap between *T. clavata* and these two species raises the possibility that competitive exclusion might restrict *T. clavata*'s realized niche. If so, *T. clavata* may have higher environmental flexibility than is suggested from its native range, which would help explain the discrepancy between its current establishment in the southeastern United States and the range predicted by our SDMs. Without any comparable large‐bodied orb weavers to the north of *T. clavata*'s introduction area, range expansion may go largely unchecked by competition as an empty niche is filled. Interestingly, southern expansion may be slowed or limited by the presence of *T. clavipes*. These species currently overlap at the edges of their distributions, so detailed study of their interactions is needed.

It is also possible that *T. clavata*'s known Asian range reflects sampling bias, with fewer observations made in southeastern Asia. Sampling bias is an inherent risk when using data from crowdsourced websites like iNaturalist (Di Cecco et al., 2021). The relative absence of *T. clavata* observations in Myanmar and Vietnam as well as the rest of continental southeastern Asia raises questions of whether differences in the number of iNaturalist users and observations across countries might bias our models. However, the extensive geographic coverage of *T. clavata*'s relatives reported in these regions, especially *N. pilipes* (over 14,000 research grade observations on iNaturalist alone), suggests the relative absence of *T. clavata* in southeastern Asia in our datasets likely represents a real pattern rather than a bias attributable to a dearth of users or observations.

Our models suggest that climates in the northeastern United States and southern Canada are more suitable matches for *T. clavata* than their current introduced range, so cooler northern climates will likely not impede continued range expansion. In spiders, natal dispersal is typically achieved by ballooning, where newly emerged spiderlings release silk and travel as aeronauts in air currents (Foelix, 1996). *Trichonephila* and *Nephila* spiders are no exception, as they are known for their long‐distance ballooning abilities (Jung et al., 2006; Kuntner & Agnarsson, 2011; Lee et al., 2015). This, paired with climatic suitability across eastern North America, may explain the observed range expansion dynamics. Our SDM predictions, coupled with *T. clavata*'s substantial powers of dispersal, limited competition with comparably sized spiders, and release from enemies in its native range, suggest continued expansion to the Northeast as an unoccupied niche is filled. Disentangling the mechanisms facilitating this range expansion is an area primed for future research.

### 4.2 Impact of *T. clavata* on native orb weaving spiders

Our study reports the first known ecological impacts of *T. clavata* in its introduced range, specifically on native orb weaving spiders. We found reduced diversity of native orbweavers in areas with prolonged presence of *T. clavata* and higher human populations; however, since these models were based on data with few *T. clavata* observations in areas with very low human population density, this conclusion must remain tentative. Future studies should experimentally test for ecologically meaningful relationships between *T. clavata* and native orb weavers to thoroughly explore the impacts of the *T. clavata* invasion to supplement our first year of correlative observational data.

Another explanation for these patterns is that *T. clavata* may be more tolerant of urban environments with greater human activity than some native species. Several spiders are known to be more commonly found in areas with a greater human impact (e.g., Damptey et al., 2022; Fraser & Frankie, 1986; Moorhead & Philpott, 2013), and some species actively thrive in these areas due to increased reproductive capacity (Lowe et al., 2014) and enhanced prey capture (Gomes, 2020). Many of the known North American non‐native spiders are synanthropic (e.g., *Pholcus phalangioides* and *P. manueli*: Campbell et al., 2020; *Cyrtophora citricola*: Chuang & Riechert, 2021; *Latrodectus geometricus*: Vetter et al., 2016; *Oecobius navus*: Voss et al., 2007), likely due to selective pressures associated with their common introduction pathway as shipping cargo stowaways (Hänggi & Straub, 2016; Nentwig, 2015). While *T. clavata* shares this proposed introduction pathway (Hoebeke et al., 2015), it is unlike the other species because it is present in both urban and natural environments in its introduced range, which increases the odds of impacts on native communities. Ultimately, more information is needed to elucidate the interactions between non‐native and native arthropods and urban areas (Gardiner et al., 2013; McIntyre, 2000) as well as the potential impacts of this invasive species as it continues to spread.

Although it has only been documented in North America for less than 15 years, *T. clavata* has already become the most locally common and abundant orb weaving spider species in our surveyed area. Its rapid spread and numerical dominance classify *T. clavata* as an invasive species (sensu Richardson, 2000). Invasive spiders can negatively impact native arthropod communities in many ways. For example, the presence of invasive *Linyphia triangularis* led to reduced densities of native spiders (Jakob et al., 2011), increased web abandonment and web building of the native *Frontinella communis*, and web takeovers of *F. communis* in Maine, United States (Bednarski et al., 2010). Candy‐striped spiders (*Enoplognatha ovata* and *E. latimana*) are known to disproportionately prey on pollinators, even actively hunting prey during pre‐dawn periods when insects are typically lethargic (Scott & McCann, 2023). We have observed *T. clavata* feeding on prey in several different arthropod groups, including cockroaches, beetles, wasps, bees, butterflies, dragonflies, grasshoppers, and other spiders (David R. Coyle, John F. Deitsch, Michael I. Sitvarin, personal observations). The high metabolic rate and quick maturation time of *T. clavata* (Davis & Frick, 2022) suggests a substantial amount of prey is taken, and thus not available to native orb weavers. Detailed studies of interactions between *T. clavata* and native orb weavers and prey are needed to better understand community responses to this invasion.

## 5 CONCLUSION

There is an abundance of suitable habitat for *T. clavata* throughout eastern North America and in some areas in the western part of the continent. *Trichonephila clavata*'s fast population expansion rate, coupled with both natural and anthropogenic dispersal mechanisms, makes it highly likely that it will continue to spread into these areas. *Trichonephila clavata* is often numerically dominant among orb weavers where it occurs and was more common and more abundant than native species in this study. Despite the nascence of *T. clavata*'s invasion, we have already found evidence of native spider biodiversity declines associated with its presence. These patterns should strongly motivate funding institutions and researchers alike to turn their attention toward this invasion and consider ways to mitigate its impacts on native communities. While impacts of *T. clavata* on human or pet health have not been documented, our data show that their ecological impacts may not be similarly benign as their invasion progresses.

## AUTHOR CONTRIBUTIONS

**David R. Nelsen:** Conceptualization (equal); data curation (equal); formal analysis (equal); investigation (lead); methodology (equal); visualization (equal); writing – original draft (equal); writing – review and editing (equal). **Aaron G. Corbit:** Formal analysis (equal); methodology (equal); visualization (equal); writing – original draft (equal); writing – review and editing (equal). **Angela Chuang:** Conceptualization (equal); methodology (equal); writing – original draft (equal); writing – review and editing (equal). **John F. Deitsch:** Conceptualization (equal); formal analysis (equal); investigation (supporting); methodology (equal); visualization (equal); writing – original draft (supporting); writing – review and editing (equal). **Michael I. Sitvarin:** Conceptualization (equal); investigation (supporting); writing – original draft (equal); writing – review and editing (equal). **David R. Coyle:** Conceptualization (equal); investigation (supporting); methodology (equal); writing – original draft (equal); writing – review and editing (equal).

## FUNDING INFORMATION

None.

## CONFLICT OF INTEREST STATEMENT

The authors have no known conflicts of interest.

## Supporting information


Appendix S1

## Data Availability

The data that support the findings of this study are openly available in Zenodo at doi: https://doi.org/10.5281/zenodo.8091991.
