# Supplementary material for: Veni, vidi, vici? Future spread and ecological impacts of a rapidly expanding invasive predator population
Source: Ecol Evol. 2023 Nov 20;13(11):e10728. doi: 10.1002/ece3.10728 (PMC10659957; doi:10.1002/ece3.10728)

Figure S1: Directed Acyclic Graph of the proposed causal relationship between predictor variables (population density, *Trichonephila clavata* presence, and their interaction) on the outcome variable (orb-weaver diversity). The outcome variable is indicated by the blue oval with an “I”, the predictor variables are indicated by the green ovals with “play” symbols, and the confounding variables are depicted as white ovals. The oval labeled “current abundance” is a mediator and therefore not included in the regression models.


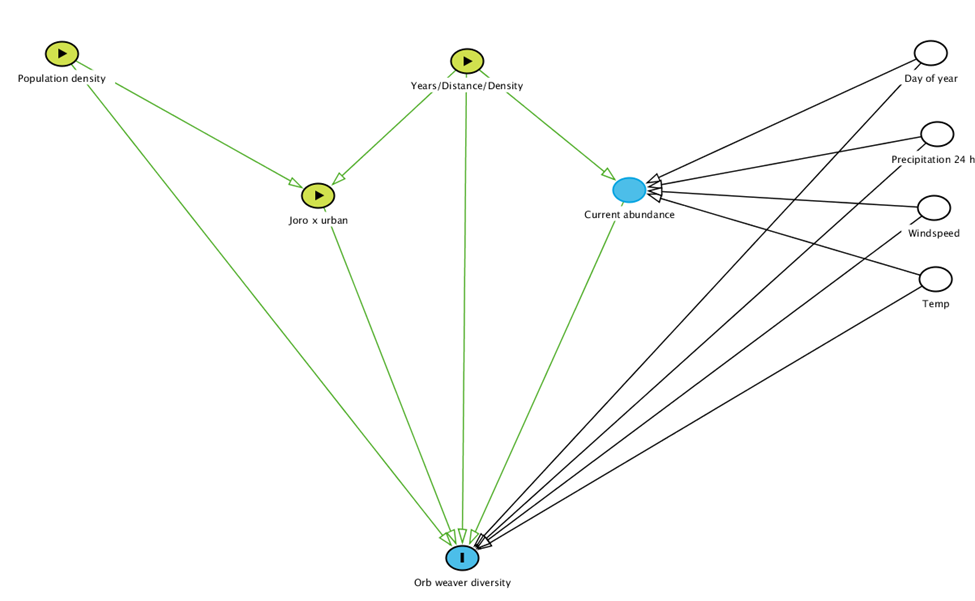


| Table S1: Results of linear and general linear models testing if the presence of *Trichinephila clavata* affects the abundance and diversity of native orb-weaving species. This table reports the coefficients, SE, P-value, and ΔAICc for all linear models: main effects not including human population density, main effects controlling for human population density, and the interaction between human population density and *T. clavata*’s historical presence. | | | | | | | | | | |
| --- | --- | --- | --- | --- | --- | --- | --- | --- | --- | --- |
| Outcome | Historic Predictor | Coefficient ± SE | | | P | | | ΔAICc | | |
|  |  | Excluding Pop | Pop Density | Interaction | Excluding Pop | Pop Density | Interaction | Excluding Pop | Pop Density | Interaction |
| Species Richness | Distance from Centroid | 4.1E-6±2.2E-6 | 3.6E-6±2.4E-6 | 2.9E-8±1.3E-8 | **0.062** | 0.14 | **0.032** | 0.39 | 2.50 | **0.00** |
|  | Years at Location | -0.059±0.044 | -0.044±0.050 | -6.6E-4±2.8E-4 | 0.17 | 0.38 | **0.016** | 2.29 | 4.16 | **0.00** |
| Shannon's | Distance from Centroid | 6.1E-6±1.9E-6 | 4.4E-6±2.1E-6 | 2.1E-8±1.0E-8 | **0.0016** | **0.037** | **0.039** | 3.16 | 2.23 | **0.00** |
|  | Years at Location | -0.099±0.037 | -0.059±0.042 | -4.0E-4±1.8E-4 | **0.0094** | 0.16 | **0.033** | 4.36 | 2.57 | **0.00** |
| Simpson's | Distance from Centroid | 2.3E-6±8.3E-7 | 1.4E-6±9.2E-7 | 9.2E-9±4.5E-9 | **0.0076** | 0.13 | **0.043** | 3.92 | 2.06 | **0.00** |
|  | Years at Location | -0.035±0.016 | -0.016±0.018 | -1.7E-4±8.0E-5 | **0.033** | 0.4 | **0.034** | 5.41 | 2.52 | **0.00** |
| Distance coefficients are in meters | | | | | | | | | | |
| Pearson’s Corr: Distance x Population: P = 3.3E-5, Pearson’s = -0.40 (-0.55 – -0.22 95% CI) | | | | | | | | | | |
| Pearson’s Corr: Years Occupied Location x Population: P = 1.2E-7, Pearson’s = 0.49 (0.33–0.63 95% CI) | | | | | | | | | | |

| Table S2: Results of Mann-Whitney U test comparing bioclimate variables between Asia and North America. | | | | |
| --- | --- | --- | --- | --- |
| Variable | *W* | *p* | *r*_rb_ | Effect size classification |
| Annual Mean Temperature | 1712432.5 | <0.0001 | -0.359 | Moderate |
| Mean Diurnal Range | 40296.5 | <0.0001 | -0.848 | Large |
| Isothermality | 443302 | <0.0001 | -0.731 | Large |
| Temperature Seasonality | 4174458 | <0.0001 | 0.36 | Moderate |
| Max Temperature of Warmest Month | 368615.5 | <0.0001 | -0.752 | Large |
| Precipitation of Warmest Quarter | 2536077 | <0.0001 | -0.119 | Small |
| Temperature Annual Range | 2708356 | <0.0001 | -0.0684 | Very Small |
| Mean Temperature of Wettest Quarter | 5850673 | <0.0001 | 0.851 | Large |
| Mean Temperature of Driest Quarter | 251011.5 | <0.0001 | -0.787 | Large |
| Mean Temperature of Warmest Quarter | 1480008.5 | <0.0001 | -0.428 | Moderate |
| Mean Temperature of Coldest Quarter | 27 | <0.0001 | -0.86 | Large |
| Annual Precipitation | 3346232 | <0.0001 | 0.118 | Small |
| Precipitation of Wettest Month | 5830043 | <0.0001 | 0.844 | Large |
| Precipitation of Driest Month | 308868.5 | <0.0001 | -0.77 | Large |
| Precipitation Seasonality | 5884104 | <0.0001 | 0.86 | Large |
| Precipitation of Wettest Quarter | 5759096.5 | <0.0001 | 0.824 | Large |
| Precipitation of Driest Quarter | 316006.5 | <0.0001 | -0.768 | Large |
| Precipitation of Warmest Quarter | 5867988.5 | <0.0001 | 0.855 | Large |
| Precipitation of Coldest Quarter | 299375 | <0.0001 | -0.772 | Large |
| Windspeed | 3972914.5 | <0.0001 | 0.302 | Moderate |
| *r*_rb_ = rank-biserial correlation coefficient | | | | |
| Very Small = < 0.1, Small = 0.1-0.3, Moderate = 0.3-0.5, & Large > 0.5 | | | | |

| Table S3: Descriptive statistics of orb-weaving spiders observed during surveys. | | | | | | |  |
| --- | --- | --- | --- | --- | --- | --- | --- |
| Species | Frequency of Location Present | Average: When Present | Frequency without Jorōs | Average: without Jorōs | Frequency with Jorōs | Average: with Jorō | Total Individuals Observed |
| *Trichonephila clavata* | 53 | 10.2 ± 1.8 | 0 | 0.0 | 53 | 10.2 ± 1.8 | 543 |
| *Micrathena mitrata* | 49 | 6.5 ± 0.85 | 27 | 6.7 ± 1.3 | 22 | 6.2 ± 1.1 | 319 |
| *Neoscona crucifera* | 44 | 1.7 ± 0.17 | 21 | 2.1 ± 0.31 | 23 | 1.4 ± 0.15 | 76 |
| *Araneus marmoreus* | 27 | 1.3 ± 0.10 | 14 | 1.1 ± 0.10 | 13 | 1.4 ± 0.18 | 34 |
| *Verrucosa arenata* | 25 | 1.4 ± 0.10 | 13 | 1.5 ± 0.14 | 12 | 1.4 ± 0.15 | 36 |
| *Leucauge venusta* | 24 | 2.8 ± 0.73 | 13 | 3.2 ± 1.3 | 11 | 2.5 ± 0.53 | 68 |
| *Neoscona domiciliorum* | 20 | 1.9 ± 0.29 | 11 | 2.0 ± 0.41 | 9 | 1.7 ± 0.44 | 37 |
| *Metepeira labyrinthea* | 14 | 2.4 ± 0.72 | 5 | 1.4 ± 0.25 | 9 | 3.0 ± 1.1 | 34 |
| *Mangora maculata* | 9 | 2.0 ± 0.53 | 6 | 1.5 ± 0.22 | 3 | 3.0 ± 1.5 | 18 |
| *Micrathena gracilis* | 8 | 1.3 ± 0.25 | 4 | 1.5 ± 0.50 | 4 | 1.0 ± 0.0 | 10 |
| *Gasteracantha cancriformis* | 8 | 2.6 ± 0.73 | 6 | 3.0 ± 0.93 | 2 | 1.5 ± 0.50 | 21 |
| *Trichonephila clavipes* | 7 | 1.1 ± 0.14 | 6 | 1.2 ± 0.17 | 1 | 1.0 ± NaC | 8 |
| *Cyclosa turbinata* | 6 | 1.3 ± 0.33 | 3 | 1.0 ± 0.0 | 3 | 1.7 ± 0.67 | 8 |
| *Micrathena sagittata* | 5 | 1.6 ± 0.25 | 2 | 1.5 ± 0.50 | 3 | 1.7 ± 0.33 | 8 |
| *Argiope aurantia* | 5 | 1.4 ± 0.40 | 1 | 1.0 ± NaC | 4 | 1.5 ± 0.50 | 7 |
| *Mecynogea lemniscata* | 3 | 3.3 ± 2.3 | 1 | 8 ± NaC | 2 | 1.0 ± 0.0 | 10 |
| Kleptoparasite | 3 | 1.3 ± 0.33 | 3 | 1.3 ± 0.33 | 0 | 0.0 | 4 |
| *Tetragnatha* sp. | 1 | 3.0 ± NaC | 1 | 3.0 ± NaC | 0 | 0.0 | 3 |
| *Mangora* sp. | 1 | 1.0 ± NaC | 1 | 1.0 ± NaC | 0 | 0.0 | 1 |
| *Uloborid* sp. | 1 | 3.0 ± NaC | 1 | 3.0 ± NaC | 0 | 0.0 | 3 |

Mean ± SE

NaC = unable to calculate

Figure S2: Interaction plots based on linear and generalized linear regression models of a location’s human population density and a measure of *Trichonephila clavata’*s presence (in space or time) on orb weaving spider diversity. Shading around trend lines represents 95% CI.


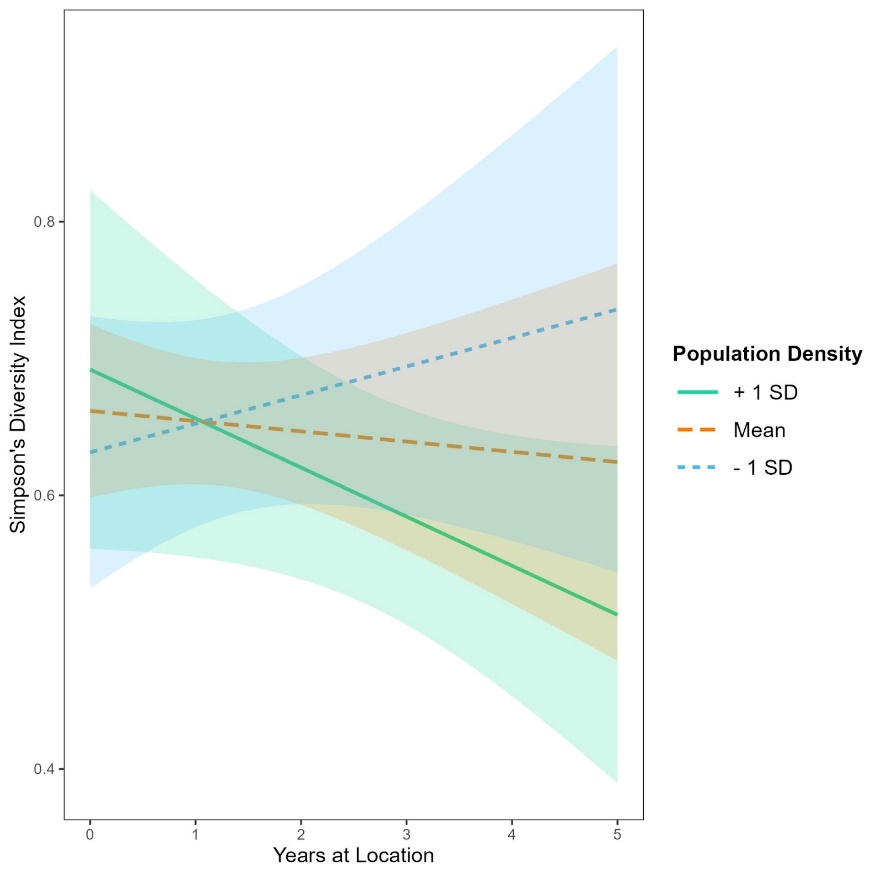

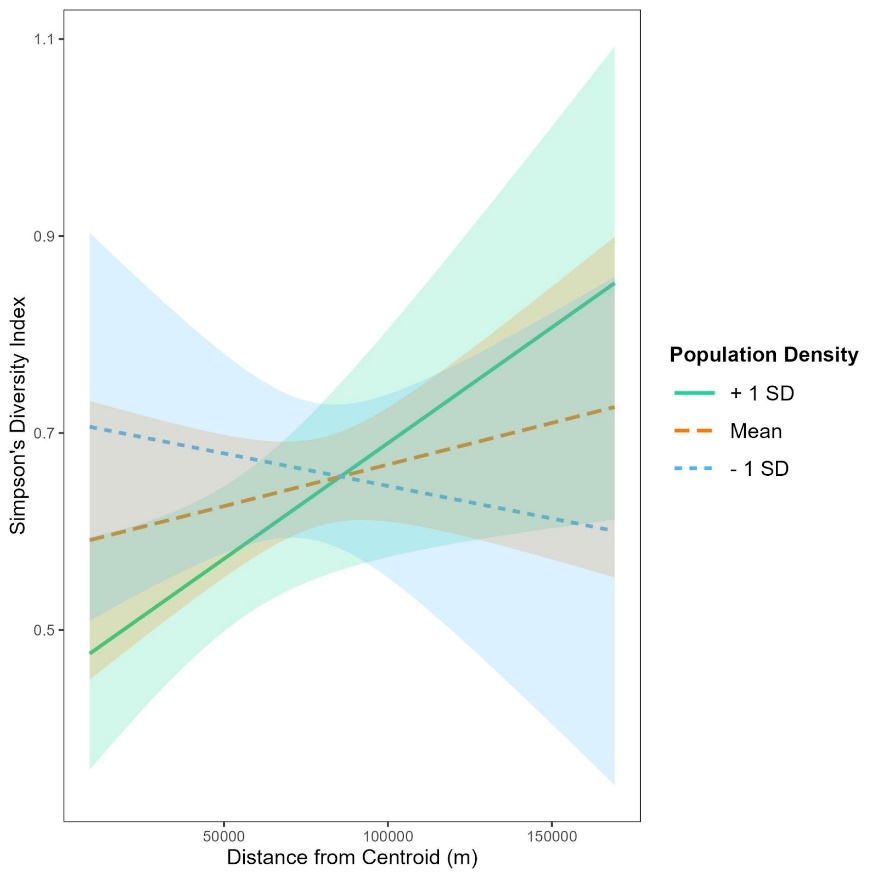

Supplement: Supplementary file 1 — Appendix S1 [file ECE3-13-e10728-s001.docx]
